# Reducing Interface Traps with High Density Hydrogen Treatment to Increase Passivated Emitter Rear Contact Cell Efficiency

**DOI:** 10.1186/s11671-019-3216-3

**Published:** 2019-12-12

**Authors:** Chih-Cheng Yang, Po-Hsun Chen, Ting-Chang Chang, Wan-Ching Su, Sung-Yu Chen, Shui-Chin Liu, Sheng-Yao Chou, Yung-Fang Tan, Chun-Chu Lin, Pei-Yu Wu, Tsung-Ming Tsai, Hui-Chun Huang

**Affiliations:** 1 Department of Materials and Optoelectronic Science, National Sun Yat-Sen University, Kaohsiung, 80424 Taiwan; 2 Department of Applied Science, R.O.C. Naval Academy, Kaohsiung, 81345 Taiwan; 3 Department of Physics, National Sun Yat-Sen University, Kaohsiung, 80424 Taiwan; 4 Green Energy & Environment Research Laboratories, Industrial Technology Research Institute, Hsinchu, 300 Taiwan

**Keywords:** High-density hydrogen treatment (HDH), Solar cell, Interface traps, SiN, Passivation layer

## Abstract

In this work, a high-density hydrogen (HDH) treatment is proposed to reduce interface traps and enhance the efficiency of the passivated emitter rear contact (PERC) device. The hydrogen gas is compressed at pressure (~ 70 atm) and relatively low temperature (~ 200 *°*C) to reduce interface traps without changing any other part of the device’s original fabrication process. Fourier-transform infrared spectroscopy (FTIR) confirmed the enhancement of Si–H bonding and secondary-ion mass spectrometry (SIMS) confirmed the SiN/Si interface traps after the HDH treatment. In addition, electrical measurements of conductance-voltage are measured and extracted to verify the interface trap density (Dit). Moreover, short circuit current density (Jsc), series resistance (Rs), and fill factor (F.F.) are analyzed with a simulated light source of 1 kW M^−2^ global AM1.5 spectrum to confirm the increase in cell efficiency. External quantum efficiency (EQE) is also measured to confirm the enhancement in conversion efficiency between different wavelengths. Finally, a model is proposed to explain the experimental result before and after the treatment.

## Introduction

Solar cells are one of many renewable energies in the world and are considered the most capable to replace transitional petrochemical energy. There are several types of solar cells based on different material systems, such as silicon [1–3], perovskite [4, 5], or III–V compounds [6, 7]. Among them, the silicon-based solar cell is commonly used for its low cost, high stability, and excellent efficiency up to 26% [8–10]. The passivated emitter rear contact (PERC) device is regarded as one of the potential devices to replace back surface field (BSF) solar cells [11, 12]. In 1983, Prof. Martin Green first proposed a PERC cell at the University of New South Wales (UNSW), the concept of which was to combine the emitter and rear passivation layer to reduce the interface defects and increase cell efficiency. Although the PERC emitter and rear passivation layer can passivate the interface defects, the film quality of either the emitter or anti-reflection coating (ARC) layer affects the interface [13–15].

According to previous work, other than improving thin film quality to reduce interface traps, post annealing treatment is another method to decrease defects [16–18]. A post treatment that of forming gas annealing in nitrogen (95%) and hydrogen (5%) at 400 °C is used to reduce interface traps with hydrogen and enhance cell efficiency. Unfortunately, such a treatment requires reaction at approximately 400 °C, a temperature too high for solar cells such as heterojunction with intrinsic thin layer (HIT) which are fabricated at temperatures under 200 °C.

In this work, we propose a suitable high-density hydrogen (HDH) treatment to reduce the interface traps between the emitter passivation layer and the n-type Si layer without the necessity to alter any additional element of device fabrication. Similar to previous research, HDH treatment is used to passivate the defects using hydrogen ions. The experimental result suggests an enhancement of the Si–H bond after the HDH treatment, according to the Fourier-transform infrared spectroscopy (FTIR) measurement secondary-ion mass spectrometry (SIMS). In addition, electrical measurements including conductance, short circuit current density (Jsc), series resistance (Rs), and fill factor (F.F.) are extracted to confirm the reduction of density of state (Dit) and the increase of cell efficiency. Finally, we also proposed a model to further illustrate the effects of HDH treatment on the PERC solar cell.

## Experimental Methods

### PERC solar cell fabrication

**Fig. 1:**
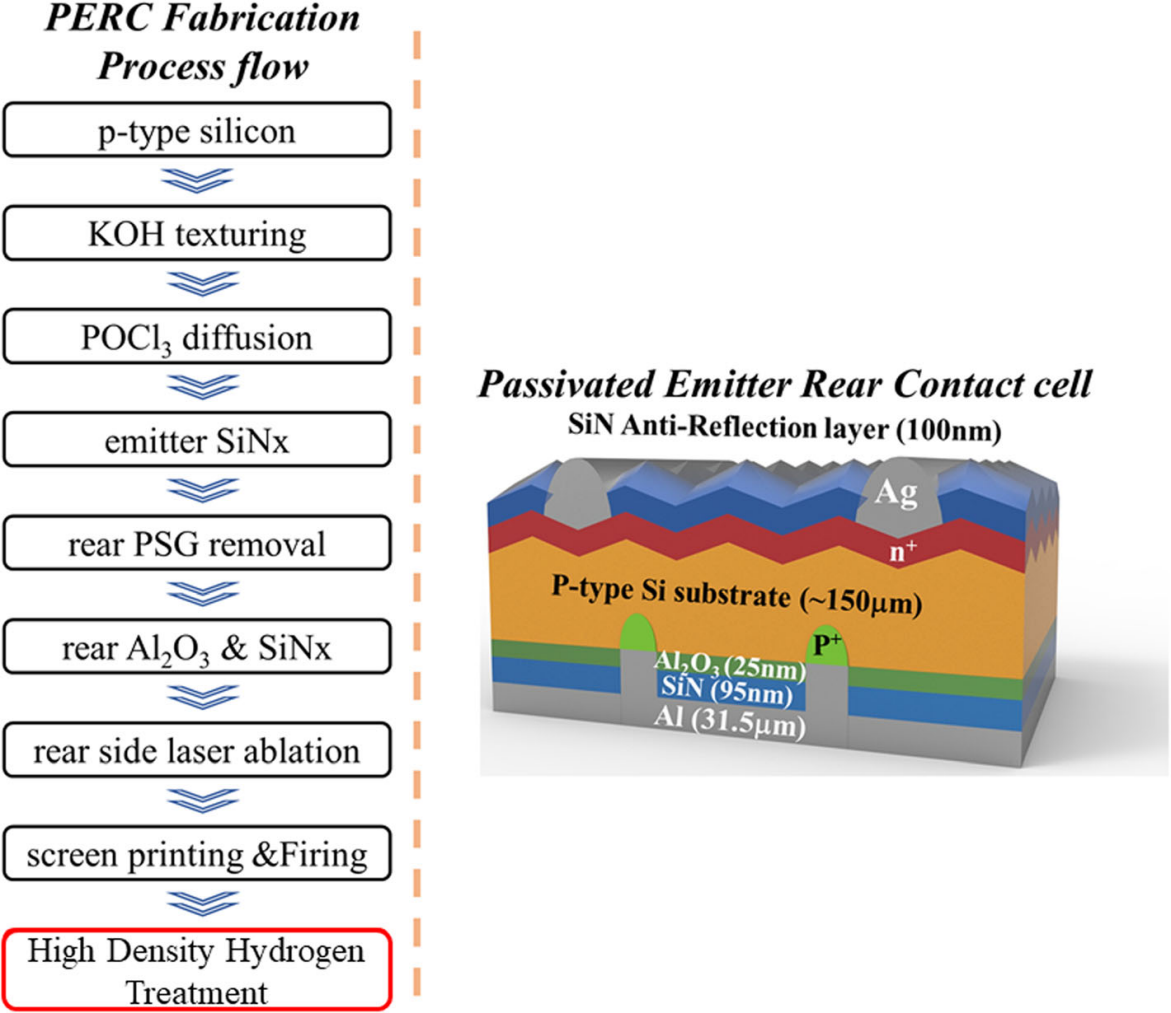
PERC fabrication process flow and passivated emitter rear contact (PERC) cell structure

The PERC fabrication process is illustrated below. The p-type Czochralski silicon is used as the substrate with a thickness of approximately 150 μm. The KOH solution is used to etch the Si substrate surface and form the pyramid-texture morphology of the surface. In order to form the p-n junction, POCl_3_ is used to diffuse into the Si substrate surface and form the n-type layer. Then, the emitter SiN passivation layer is deposited via chemical vapor deposition (CVD) as an anti-reflection coating (ARC) layer. After the ARC layer is deposited, HF solution is used to remove the rear side n-type layer. Then, the Al_2_O_3_ layer is deposited as the rear passivation layer with a thickness of 25 nm by atomic layer deposition (ALD). The 95-nm-thick SiN layer is then deposited by CVD. After the rear passivation process is finished, laser ablation is applied to cut grooves for the preparation of the screen printing process of the silver (Ag) top electrode used on the ARC layer, while aluminum (Al) is used for the bottom electrode. Finally, the device is heated in a firing process in order to ensure proper contact between metal and semiconductor. The structure of the PERC device is shown in Fig. 1.

### HDH Treatment

**Fig. 2:**
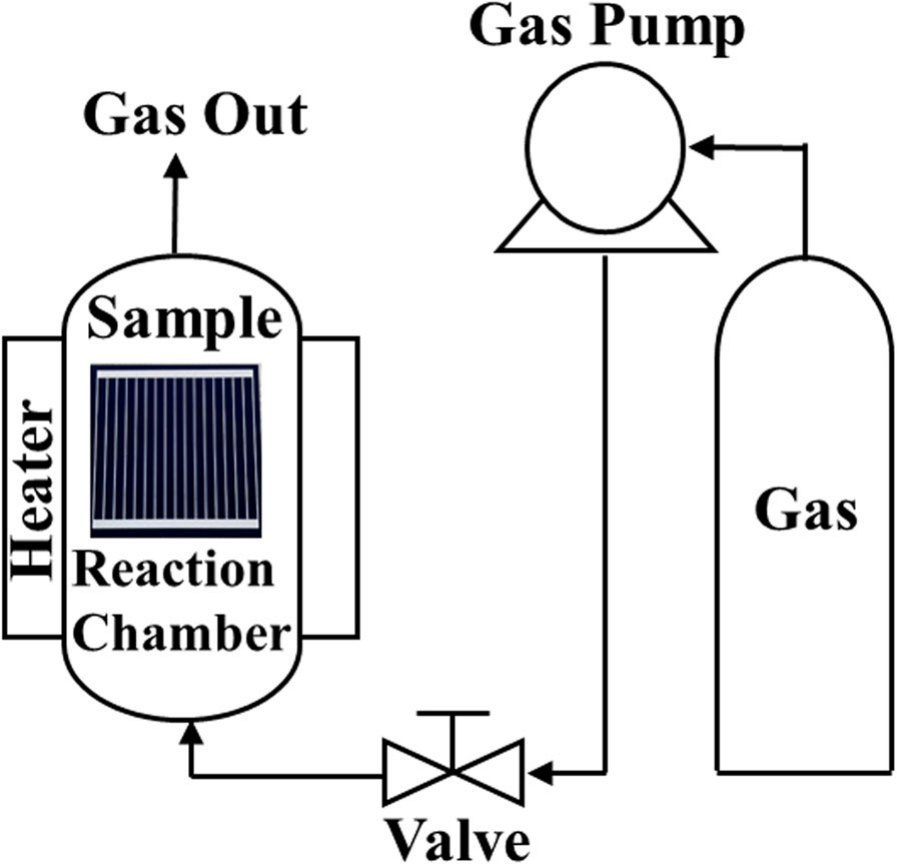
High-density hydrogen (HDH) treatment process flow

The HDH treatment is then applied to the PERC device. The process of HDH treatment is as depicted in Fig. 2. The hydrogen gas is used as the treatment source and is pumped into the reaction chamber containing the PERC device. Then, the gas is compressed to 70 atm and the reaction temperature is set at 200 °C for 1 h. The gas is then pumped out to finish the HDH treatment.

### Material Characterization

Bruker VERTEX 70v FTIR is used to analyze Si–H bonding before and after treatment and ION-TOF, TOF-SIMS V is used to analyze hydrogen ratio at SiN/Si interface.

### Electrical Characterization

I-V and G-V characteristics are measured with an Agilent B1500 semiconductor analyzer and Cascade M150 probe station in a dark box for both light and dark conditions. The parameters of efficiency (Jsc, Rs, and fill factor) are extracted at a simulated light source of 1 kW M^−2^ global AM1.5 spectrum at 25 °C. QEX10 Solar Cell external quantum efficiency (EQE) is used to analyze the efficiency from 300 to 1200 nm.

## Result and Discussion

**Fig. 3:**
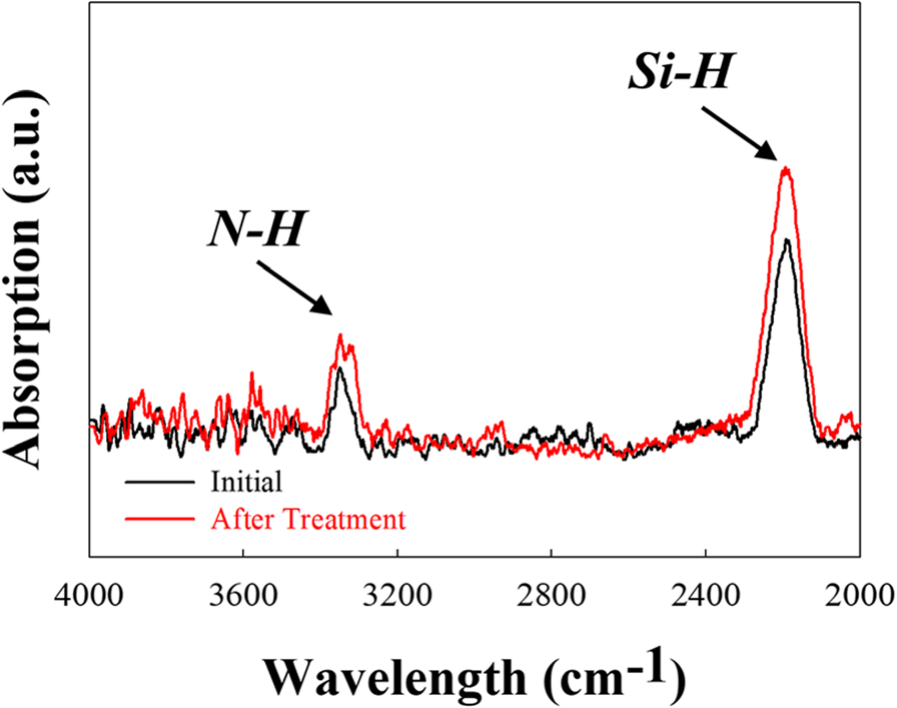
Emitter SiN passivation layer thin film measured with FTIR spectrum

The emitter SiN passivation layer with and without the HDH treatment is examined using a FTIR analysis. As shown in Fig. 3, the SiN with and with HDH treatment both exhibit 3350 cm^−1^ of the N–H stretching bond and 2165 cm^−1^ of the Si–H stretching bond [19–21]. However, the absorption peak intensity ratios of N–H and Si–H bonding are both enhanced after the treatment, which implies that hydrogen is injected into the SiN layer.

**Fig. 4:**
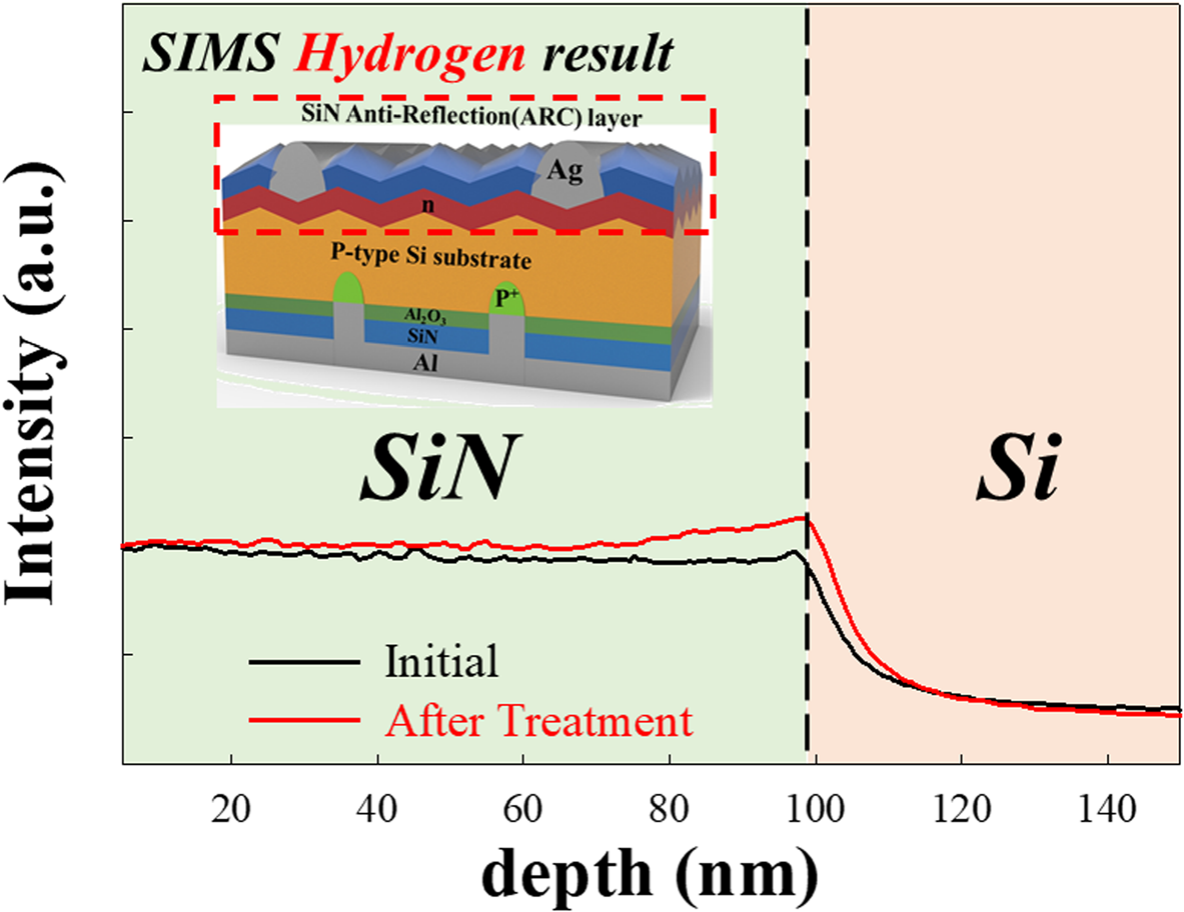
Emitter SiN passivation layer thin film measured with SIMS

In order to confirm that the HDH treatment reduces the SiN/Si interface traps, secondary-ion mass spectrometry (SIMS) is used to confirm the hydrogen distribution [22, 23]. In Fig. 4, because the SiN layer is deposited using CVD, the hydrogen intensity in this SiN layer is higher than in the Si. After the treatment, while the hydrogen intensity is not obviously increased in bulk, the intensity is clearly enhanced at the SiN and Si interface, and this result indicates that the HDH treatment reacts at the SiN/Si interface.

**Fig. 5:**
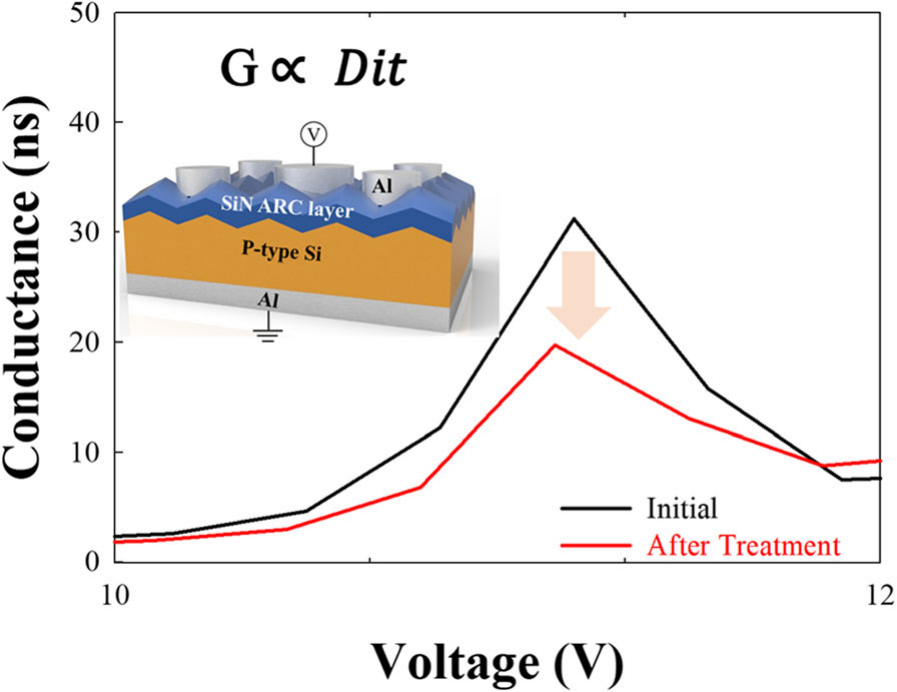
Al/SiN/p-Si/Al device is analyzed by conductance-voltage characteristic with interface traps

To further confirm the difference in Dit between the emitter SiN layer and p-Si substrate after HDH treatment in Fig. 5, the Al/SiN/p-Si/Al metal-insulator-semiconductor (MIS) structure is fabricated. Since the SiN and p-Si interface has a large amount of defects, the G-V result can be applied to extract the interface trap density (Dit) [24]. The conductance equation is given as:

1 $$ \frac{Gp}{\omega }=\frac{D_{it}\omega {\tau}_{it}}{1+{\omega}^2{\tau}_{it}^2} $$

where *ω* is angular frequency, *τ* is the carrier lifetime, and *Gp* is the frequency-dependent conductance. To simplify Eq. 1, the Dit is related to conductance, and the conductance peak is reduced after the treatment, which suggests the HDH treatment can reduce the PERC interface traps.

**Fig. 6:**
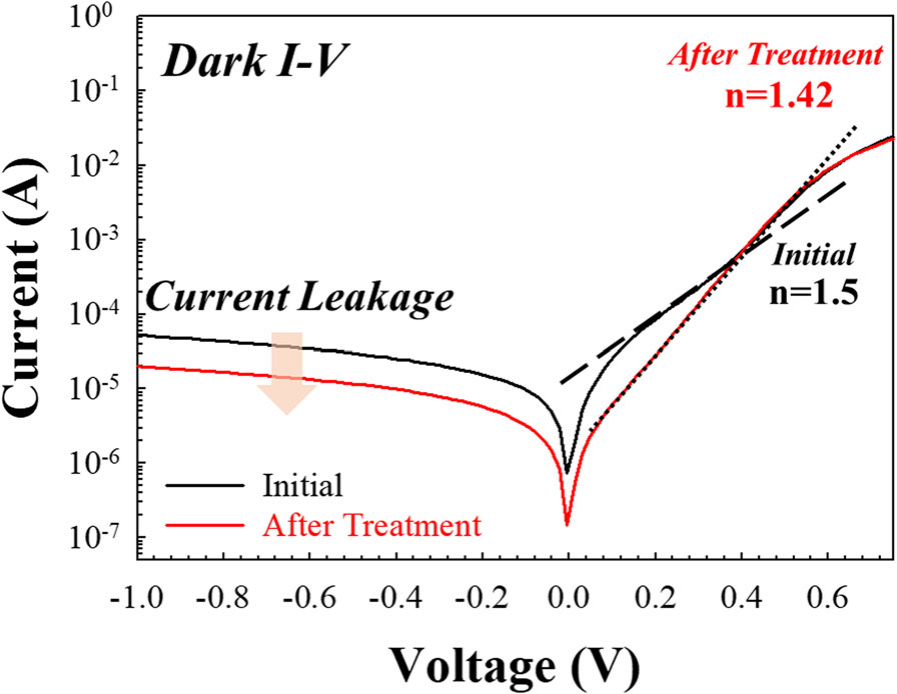
Analysis of I-V characteristics under dark conditions with current leakage and ideal factor.

Next, electrical measurements are carried out on the PERC cell device in both light and dark conditions. The device bias is applied to the Al bottom electrode, while the top electrode is ground. The sweeping range of the voltage is from − 1 to 0.75 V. Figure 6 shows the I-V characteristic under dark conditions. The current leakage is reduced significantly after HDH treatment, with the ratio of the decrease being about 0.5 orders. In addition, on the right side of the I-V characteristic, the hump of the on current is found to be reduced after the treatment. We also extract the I-V curve and convert it as the ideal factor following the diode current equation:

2 $$ I={I}_s\left[\mathit{\exp}\left(\frac{qV}{nkT}\right)-1\right] $$

where *I*_*s*_ is the saturation current, *q* is the electronic charge, *V* is the applied voltage, *n* is the ideal factor, *k* is the Boltzmann constant, and *T* is the absolute temperature. Equation 2 can be further simplified into $I={I}_s\left[\mathit{\exp}\left(\frac{qV}{nkT}\right)-1\right]$; when the *n* value is close to 1, the on current is close to the diffusion current. When the *n* value is close to 2, this means that the on current is close to the combination current [25]. After the treatment, the slope of the on current is reduced from 1.5 to 1.42, which means that the on current is close to the diffusion current after treatment due to the decrease in the number of defects.

**Fig. 7:**
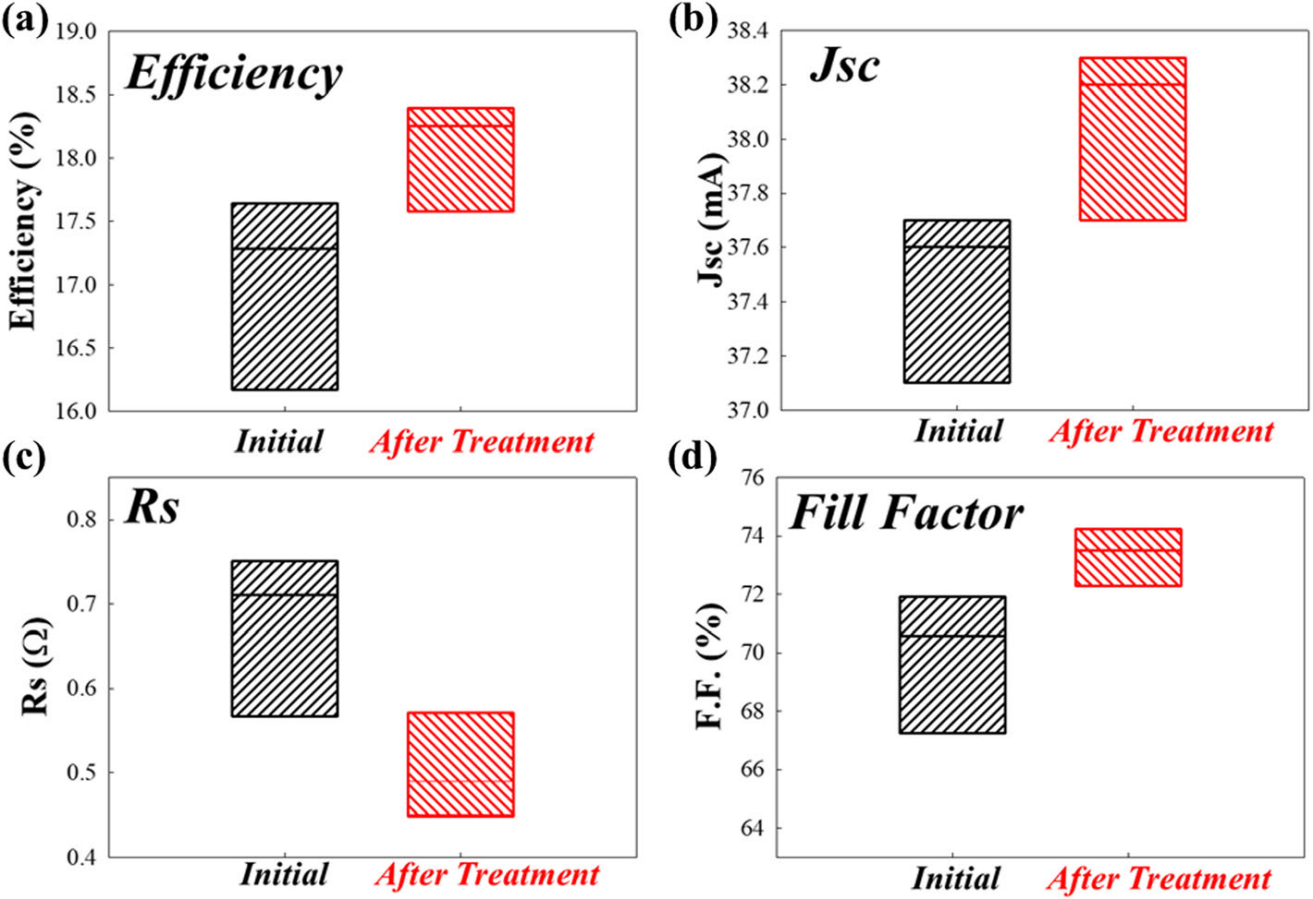
PERC I-V characteristics for **a** efficiency, **b** short circuit current density (Jsc), **c** series resistance (Rs), and **d** fill factor (F.F.)

To further examine the electrical characteristics, the parameters of efficiency (Jsc, Rs, and fill factor) are extracted at a simulated light source of 1 kW M^−2^ global AM1.5 spectrum at 25 °C. After the HDH treatment, the average efficiency is enhanced from 17.3 to 18.2%, as shown in Fig. 7a. The Jsc also increases from 37.6 to 38.2 mA, as shown in Fig. 7b. In addition, the Rs has been reduced from 0.712 to 0.487 after treatment, as in Fig. 7c. As for the fill factor, it increases from 70.5 to 73.3, as shown in Fig. 7d.

**Fig. 8:**
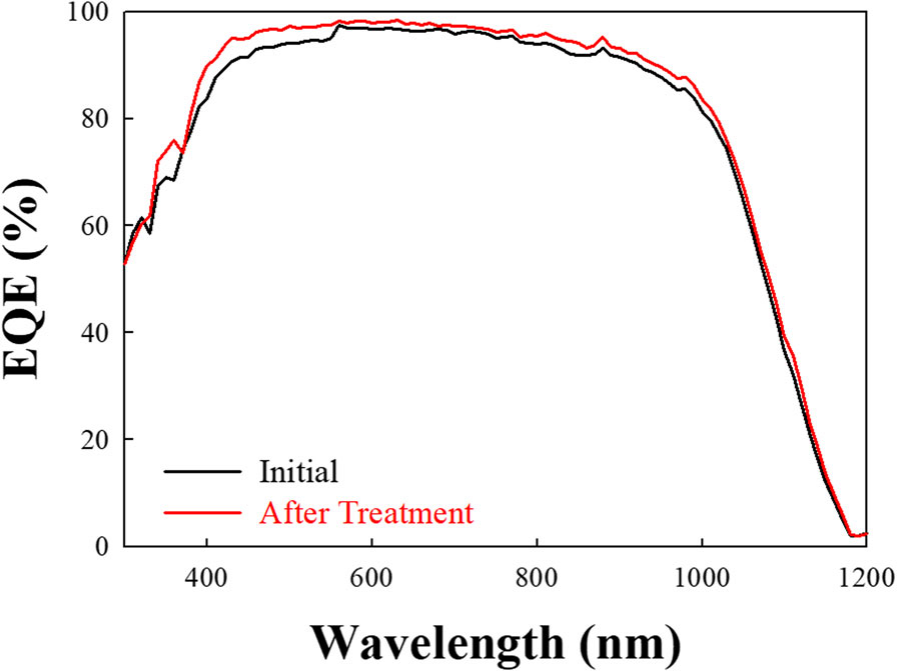
External quantum efficiency (EQE) is measured from 300 to 1200 nm

In order to confirm the conversion efficiency at different wavelength ranges, the external quantum efficiency (EQE) is used to analyze the wavelength from 300 to 1200 nm [26, 27]. As shown in Fig. 8, the quantum efficiency before HDH treatment has an average EQE of 94% between 400 and 600 nm. However, after the HDH treatment, we can obtain an even higher EQE result. The results show an increase to 97% between 400 and 600 nm, which is induced by the suppression of the emitter SiN/Si interface traps.

**Fig. 9:**
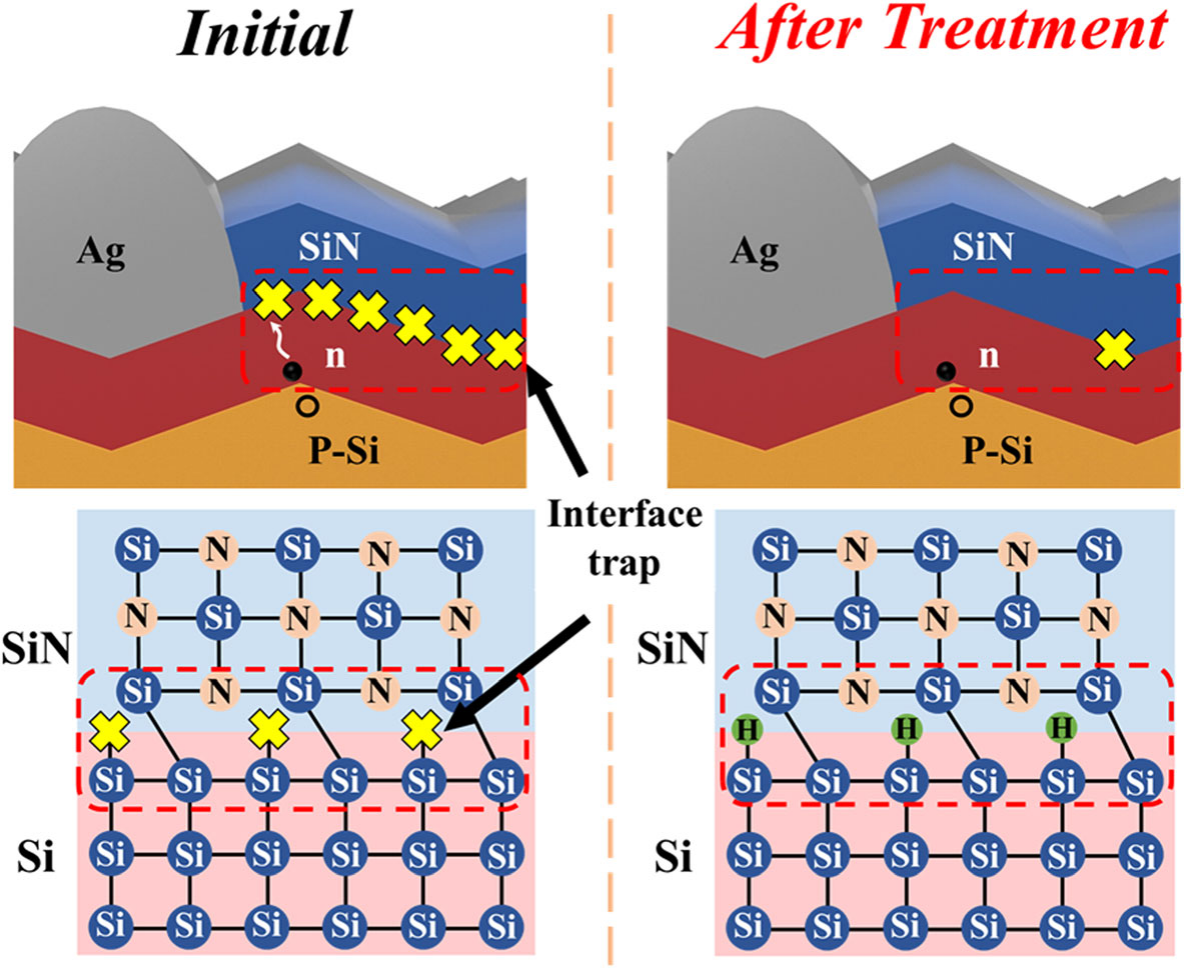
PERC emitter of Ag/SiN/n-type Si structure and SiN/Si interface trap structure at initial and after treatment

Finally, we propose a model to explain the effects of HDH on the PERC device. The PERC emitter of Ag/SiN/n-type Si structure and the relationship to SiN/Si interface trap structure are demonstrated in Fig. 9. When the electron-hole pair is generated in the p-n junction, induced by light, the electron moves to the Ag top electrode. If there are interface traps at the SiN/Si interface, they will assist electron recombination with holes. To reduce the interface traps, HDH treatment is applied to the PERC device, with high-pressure gas being used to injected hydrogen into the device and react with the interface. After the treatment, hydrogen bonds with the dangling bond at the SiN/Si interface and interface traps are reduced. Therefore, recombination decreases, which reduces current leakage and enhances the Jsc and cell efficiency.

## Conclusion

In this study, HDH treatment is successfully proposed to reduce interface traps and enhance device efficiency. The FTIR spectrum shows that Si–H bonding is enhanced and conductance-voltage peak decreases after the treatment. Therefore, the reduced number of interface traps leads to a reduction in current leakage, and the ideal factor value is also decreased. Moreover, the efficiency is enhanced after the treatment, and Jsc, Rs, and fill factor are increased. Finally, the EQE result demonstrates an enhancement of short wavelength, which is evidence of a reduction in emitter interface traps.

## Data Availability

All data is available from the authors via a reasonable request.

## Abbreviations

HDH: High-density hydrogen treatment
PERC: Passivated emitter rear contact cell
FTIR: Fourier-transform infrared spectroscopy
SIMS: Secondary-ion mass spectrometry
Dit: Interface trap density
Jsc: Circuit current density
Rs: Series resistance
F.F.: Fill factor
EQE: External quantum efficiency
BSF: Back surface field
UNSW: University of New South Wales
ARC: Anti-reflection coating
CVD: Chemical vapor deposition
ALD: Atomic layer deposition
MIS: Metal-insulator-semiconductor structure
